# Development of a novel combined nomogram model integrating deep learning radiomics to diagnose IgA nephropathy clinically

**DOI:** 10.1080/0886022X.2023.2271104

**Published:** 2023-10-20

**Authors:** Xiachuan Qin, Linlin Xia, Qianqing Ma, Dongliang Cheng, Chaoxue Zhang

**Affiliations:** a Department of Ultrasound, Nanchong Central Hospital, The Second Clinical Medical College, North Sichuan Medical College (University), Nan Chong, Sichuan Province, China; b Department of Ultrasound, The First Affiliated Hospital of Anhui Medical University, Hefei, Anhui Province, China; c Hebin Intelligent Robots Co., LTD, Hefei, Anhui Province, China

**Keywords:** IgA nephropathy, ultrasound, superb microvascular imaging, deep learning, radiomics

## Abstract

This study aimed to develop and validate a combined nomogram model based on superb microvascular imaging (SMI)-based deep learning (DL), radiomics characteristics, and clinical factors for noninvasive differentiation between immunoglobulin A nephropathy (IgAN) and non-IgAN.We prospectively enrolled patients with chronic kidney disease who underwent renal biopsy from May 2022 to December 2022 and performed an ultrasound and SMI the day before renal biopsy. The selected patients were randomly divided into training and testing cohorts in a 7:3 ratio. We extracted DL and radiometric features from the two-dimensional ultrasound and SMI images. A combined nomograph model was developed by combining the predictive probability of DL with clinical factors using multivariate logistic regression analysis. The proposed model’s utility was evaluated using receiver operating characteristics, calibration, and decision curve analysis. In this study, 120 patients with primary glomerular disease were included, including 84 in the training and 36 in the test cohorts. In the testing cohort, the ROC of the radiomics model was 0.816 (95% CI:0.663–0.968), and the ROC of the DL model was 0.844 (95% CI:0.717–0.971). The nomogram model combined with independent clinical risk factors (IgA and hematuria) showed strong discrimination, with an ROC of 0.884 (95% CI:0.773–0.996) in the testing cohort. Decision curve analysis verified the clinical practicability of the combined nomogram. The combined nomogram model based on SMI can accurately and noninvasively distinguish IgAN from non-IgAN and help physicians make clearer patient treatment plans.

## Introduction

Immunoglobulin A (IgA) nephropathy (IgAN) is the most common form of primary glomerulonephritis worldwide, accounting for more than 40% of all biopsies in China, and is the main cause of chronic kidney disease (CKD) and renal failure [1–3]. The IgAN typically manifests as asymptomatic seizures, accompanied by proteinuria, hypoalbuminemia, edema, and hyperlipidemia.However, these symptoms are neither suitable for the diagnosis of primary glomerulopathy [4]. Renal biopsy is the gold standard for diagnosing IgAN. There are many disadvantages to renal biopsy. For example, as an invasive examination, renal biopsy may cause multiple complications such as perirenal hematoma, pain, and infection [5–7]. Due to the limitations of puncture technology, some patients fear and reject renal biopsy or develop puncture contraindications; these and other reasons result in the biopsy not being conducted normally and, subsequently, hinder patients from obtaining the best treatment. Therefore, there is an urgent need for a simple and noninvasive IgAN diagnosis model. Although some studies have attempted to make noninvasive IgAN and non-IgAN diagnoses preoperatively, diagnostic efficiency remains low [8–11]

The well-known advantages of ultrasound imaging, such as low cost, noninvasiveness, and lack of ionizing radiation, make it an attractive choice for detecting kidney diseases [6]. Ultrasound imaging mainly comprises two basic modes: B-mode ultrasound (BUS) and color Doppler flow imaging (CDFI). Recently, superb microvascular imaging (SMI) has been applied as a relatively new noninvasive flow imaging mode, which uses an adaptive wall filter different from CDFI and minimizes flash artifacts. Even without any contrast agent, SMI can also improve slow-flow visibility and sensitivity of small vessel signal detection [12,13]. Compared with traditional color flow imaging, SMI is more sensitive in detecting low-velocity blood flow and displays more microvascular information [14–16]. It also shows good consistency with contrast-enhanced ultrasound(CEUS) [16–18]. To a large extent, ultrasound’s diagnostic performance depends on radiologists’ clinical and professional knowledge. Subjective image interpretation and lack of effective quantification are the main difficulties ultrasound diagnosis faces [19].

Artificial intelligence is widely used because of its excellent performance in image-recognition tasks. It can effectively improve the diagnostic accuracy of medical image interpretation and increase the objectivity of diagnosis [20,21]. The conversion of medical images into digital high-throughput quantitative features in radiomics has received increased attention [22,23]. Recently, we found that radiomics can effectively identify subclinical changes, which may be biomarkers, using ultrasound images [24,25]. Deep learning (DL) can directly couple feature extraction, feature selection, and prediction model construction into a neural network model through end-to-end learning from medical images, thus greatly simplifying the process of radiomics analysis [26,27]. The combination of DL classification networks and radiomics frameworks in integrated systems has become an emerging trend in achieving good performance in clinical tasks [28–30]. Based on the above, we established a new combined nomogram model combining DL, radiomics, and clinical indicators based on SMI imaging. We tried to distinguish IgAN from non-IgAN without histopathological data noninvasively.

## Materials and methods

### Patients

This prospective research conformed to the guidelines of the Declaration of Helsinki and was approved by the Internal Review Committee (PJ2022-11-29). Written informed consent was obtained from each study participant.

**Figure 1.**
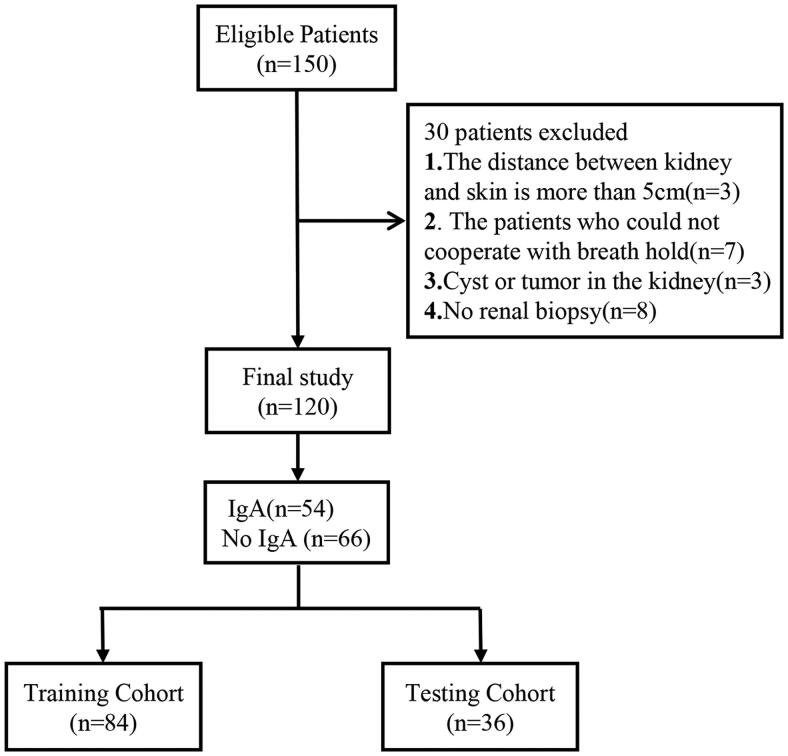
The patient Recruitment pathway and the inclusion and exclusion criteria.

We continuously recruited 120 patients with primary glomerular disease confirmed by biopsy as research subjects between May 2022 and December 2022. We assigned them to two cohorts: the IgAN and the non-IgAN cohorts. Renal biopsy was performed within 3 days after renal ultrasound measurement by two experienced nephrologists. The right kidney was selected for renal biopsy. The inclusion criteria were as follows: 1) the diagnosis of primary glomerular disease confirmed by renal puncture biopsy of at least ten glomeruli in the specimen visible under a light microscope; 2) age >18 years; and 3) < 5 cm distance between the kidney and the skin. The exclusion criteria were as follows: 1) secondary glomerular disease; 2) diagnosis of infection, tumor, or autoimmune disease; 3) renal artery stenosis or urinary tract obstruction; and 4) presence of kidney cysts or tumor. The process for case selection is shown in Figure 1. Lesions in the development dataset were randomly assigned to a training cohort (70%) or a testing cohort (30%).

We collected data on age, sex, mean blood pressure, red blood cell count, white blood cell count, hemoglobin (Hgb) levels, platelet count, neutrophil percentage, alanine aminotransferase (ALT) levels, aspartate aminotransferase (AST) levels, alkaline phosphatase (ALP) levels, total protein (TP) levels, albumin (ALB) levels, lactate dehydrogenase (LDH) levels, creatinine (CRE) levels, uric acid (UA) levels, estimated glomerular filtration rate (eGFR), urea (UREA), total cholesterol (TC) levels, triglyceride (TG) levels, high-density lipoprotein cholesterol (HDL-C) levels, low-density lipoprotein cholesterol (LDL-C) levels, immunoglobulin G (IgG) levels, IgA levels, immunoglobulin M (IgM) levels, complement C3, urine occult blood, and 24-h urine protein.

Single-factor logistic regression analysis was used to analyze the correlation between clinical parameters and IgAN. Then multi-factor logistic regression analysis was performed on the relevant factors (*p* < 0.05) obtained from the previous single-factor regression analysis to determine the independent predictive factors significantly related to IgAN.

### Ultrasonic examination

An experienced ultrasound physician (with 16 years of experience in renal ultrasound) performed image acquisition on the day before the patient underwent renal puncture using a Canon ultrasonic system Aplio 700 (Canon Medical Systems, Otawara, Japan) with a 3.5 MHz linear probe (i8C1) during fasting for more than 6 h and apnea at the end of inspiration. In all the participants, the right kidney was examined by ultrasound, having undergone right kidney biopsies. With patients lying on the left side, the ultrasound probe was gently positioned in the right abdomen by oblique projection, and the 3.5 MHz probe was used for kidney ultrasound examination. The probe was placed on the posterior axillary line, the position and angle of the probe were adjusted to obtain a longitudinal image of the kidney, and the two-dimensional image was consecutive collected.

The ultrasound scanner was then switched to monochrome SMI mode. The SMI-specific area of interest frame was placed on the whole kidney. The mechanical index was 1.6, the frame rate was 25–35 frames/s, the dynamic range was 65–75 dB, and the SMI speed was 3.5 cm/s. After obtaining the optimal blood flow section of the kidney, the images were continuously collected.

### Radiomics feature extraction

The kidney region of interest on ultrasound was manually segmented using ITK software (version 3.8.0, http://www.itksnap.org/pmwiki/pmwiki.php?n=Downloads.SNAP3). Then radiomics feature extraction was conducted using Pyradimics (version 2.2.0, http://www.radiomics.io/pyradiomics.htm), which could extract high-throughput features from ultrasonic images using various hard-coded feature algorithms. To assess the repeatability of radiomics features, the intraclass correlation efficiency (ICC) was calculated from ultrasound images of 50 randomly selected patients. Two sonographers experienced in abdominal ultrasound imaging, sonographer 1 (with 9 years of experience) and sonographer 2 (with 7 years of experience), performed the repeated ROI segmentation blinding to each other. Then the inter-group consistency of features was evaluated. After a month, sonographer 1 repeated the segmentation task to evaluate the intra-group consistency of features. The feature with an ICC value > 0.75 was considered to have high repeatability. Sonographer 1 performed the remaining image segmentation task alone.

The process of radiomics features selection consists of several steps. Firstly, features with an ICC > 0.75 in the training cohort were selected. Secondly, the Mann-Whitney *U*-test was utilized to identify features that exhibited significant differences between the IgAN and non-IgAN groups in the training cohort. Thirdly, the Spearman correlation coefficient was calculated to evaluate the relationship between each pair of features, and if the correlation coefficient was > 0.9, only the features with a higher AUC were retained. Finally, the LASSO algorithm was implemented to reduce the dimension of features, and the features with non-zero coefficients were selected. The entire pipeline integrated statistical testing methods and robust feature selection algorithms to identify the most significant features for subsequent model construction.

### Deep learning feature extraction

Before input the images into the deep learning framework, a sonographer who was experienced in kidney ultrasound cropped all the images to contain the entire kidney mask area. The images with the largest ROI of the kidney were then cropped. The cropped kidney mask maintained the complete edge without exceeding the image boundary. Additionally, ultrasound images might be noisy and contain artifacts interfering deep learning process, so we use a traditional wavelet-based denoising algorithm to reduce noise and enhance image features. All the ultrasound images were then resized to a fixed size of 224 × 224 pixels, and the pixel values were normalized to a fixed range of [0, 1] through the z-score method, to improve consistency and reduce complexity. Before the deep learning model training, we performed image augmentation in all the selected images, including rotation (90°, 180°, and 270°), flipping (horizontal and vertical), and color jittering, to meet the need for data diversity in the real world. After image augmentation, additional training data were generated from original images and could improve the robustness of the deep learning model. It also prevented model overfitting and improved the generalization performance of the deep learning model.

For our task, we constructed a specific siamese neural network, which consisted of two identical neural networks that shared the same weights. Those two networks are fed with two datasets (US images and SMI images) for the same classification task. ResNet101 has been proven to be a feature extraction tool with good stability and performance [31]. It has 101 layers, including convolutional, pooling, activation, and batch normalization layers. The ResNet-101 was used as the base model for feature extraction in our siamese neural network. To avoid model overfitting and learning the important features from input images, skip connections were used to learn residual mappings and allow gradients to flow more easily through the network. Transfer learning is used to reduce training time and improve model performance in new tasks by transferring parameters from similar tasks that have already been trained. Since our image dataset was relatively small, a transfer learning strategy was used in our siamese neural network. We collected the ImageNet dataset, which contains millions of images in thousands of classes and has achieved great performance in classification tasks, to pre-train the ResNet-101 model. Then a total of 1102 SMI images and 1105 US images were input as original images in the pre-trained model for model training.

During the transfer learning process, the pre-trained weights of the ResNet-101 model are fine-tuned on altrasound images dataset for our calssification task. Considering the front layers had learned general features that were adapted to many tasks, we freezed the weights of the pre-trained front layers. We trained the last ten layers of the ResNet-101 network so that the later layers could learn more specific and complex features that were more task-dependent. The learning rate was set as 0.001 to control the step size during the gradient descent optimization process. To ensure a relatively faster convergence, the batch size was set as 64 during model training. We also used the regularization methods including dropout and weight decay, which help prevent overfitting of our model. A dropout rate of 0.1 and a weight decay of 0.0001 were used in the ResNet-101 model. The number of epochs in our study was set as 200, which determined the times the model iterated on the entire ultrasound images dataset. We collected the feature vectors in the average-pooling layer of the model as the deep learning features and used the ensemble learning method to calculate the average feature value for each patient. Finally, 2048 SMI-based and 2048 US-based deep learning features were obtained. The image dataset of the testing cohort was input into the trained model to extract deep learning features for further analysis as well.

### Establishment of clinical nomogram model for DL radiomics

**Figure 2.**
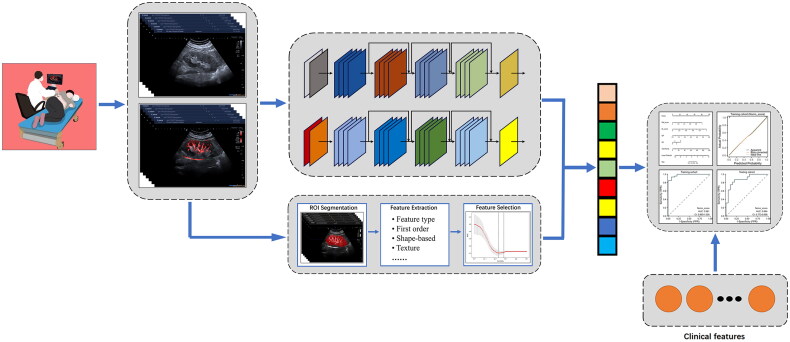
The flow chart of the combined nomogram model integrating deep learning radiomics.

In our study, several feature selection methods were utilized to select optimal features, including the ICC method, *U*-test, LASSO (minimum absolute shrinkage and selection operator) with 10-fold cross-testing. In the training cohort, we performed feature selection for radiomics features and deep learning features, respectively. Two support vector machine (SVM) machine learning models were trained to construct radiomics model (Rad_model) and a deep learning model (DL_model). These two models’ output scores were combined with clinical characteristics for further univariate and multivariate logistic regression analysis. A combined nomogram was then developed based on the significant clinical characteristics, Rad_model score, and DL_model score. The calibration curve was used to evaluate the calibration of the nomogram. The workflow of our study is shown in Figure 2.

### Statistical analysis

All statistical analyses were performed using SPSS (version 25.0; IBM Corp., Armonk, NY, USA) and Python 2.7 (Python Software Foundation, Beaverton, OR, USA). Quantitative data with normal distribution are expressed as mean ± standard deviation, while quantitative data with non-normal distribution are expressed as median ± interquartile interval. At the same time, the classification variables are expressed in numbers and percentages. The chi-square test, two independent samples, Student’s *t*-test, and Mann-Whitney *U* test were used for univariate analysis. Statistical significance was set at *p* < 0.05. The diagnostic performances of the Rad_model, the DL_model, and the combining nomogram were evaluated based on the area under the curve (AUC). To evaluate the clinical effectiveness of the models, decision curve analysis (DCA) was performed by calculating the net benefit in different probability thresholds of the training and testing cohorts.

## Results

Patient and baseline characteristics are shown in Table 1. This study recruited 120 patients with primary glomerular disease. A total of 3060 ultrasound images were obtained, including 1530 for SMI and 1530 for US. Among the patients, 54 cases had IgAN, and 66 cases had non-IgAN, which consisted of 30 cases of membranous nephropathy, 10 cases of minimal degenerative nephropathy, 5 cases of glomeruloid cell disease, 3 cases of focal segmental glomerulosclerosis, and 18 cases of mesangial proliferative glomerulonephritis. In the training cohort, a total of 2204 images were obtained, including 1102 images for SMI and 1202 images for US.38 of 84 patients (45.2%) had IgAN, and 46 of 84 patients (54.8%) had non-IgAN, as determined by pathology. In the testing cohort, a total of 856 images were obtained, including 428 images for SMI and 428 images for US.16 of 36 patients (44.4%) had IgAN, and 20 of 36 patients (55.6%) had non-IgAN. There was no group difference of baseline characteristics between the training cohort and the testing cohort.

**Table 1.** Patients’ baseline clinical characteristics.

	Training cohort		Testing cohort	
clinical characteristics	IgAN	Non-IgAN	IgAN	Non-IgAN
Sex(male/female)	14/24	22/24	7/9	12/8
Age	39.1 ± 13	44.7 ± 11.9	37.3 ± 10.4	46.1 ± 17.4
Renal length(cm)	103.7 ± 7.7	108.8 ± 9.7	102.1 ± 8.7	107.1 ± 9.5
RI of MRA	0.68 ± 0.06	0.66 ± 0.06	0.66 ± 0.04	0.67 ± 0.07
Average pressure(mmHg)	100 ± 12.8	103.6 ± 12.9	106.4 ± 11.2	94.8 ± 11.7
CRE(umol/L)	110.6 ± 95	88.6 ± 44	96 ± 43.7	75.9 ± 23.9
eGFR(ml/min.1.73m^2^)	82.3 ± 33.3	93.2 ± 30.4	84.9 ± 34.2	102.6 ± 27.8
IgA(g/L)	3.2 ± 1.2	2.2 ± 1	3.05 ± 0.8	2.2 ± 1.06
IgG(g/L)	11.3 ± 2	9.3 ± 3.6	11.3 ± 3.1	6.8 ± 2.9
24H urinary protein (g/24h)	1.29 ± 1	3.9 ± 4.5	1.58 ± 3	5.1 ± 4

MRA: main renal artery; RI: resistance index.

### Establishment of clinical predictors

Univariate analysis of the training cohort showed that the following 11 items were significantly associated with IgAN (*p* < 0.05): AST (*p* = 0.047), TP (*p* = 0.007), ALB (*p* = 0.003), LDH (*p* = 0.006), TC (*p* = 0.000), LDL-C (*p* = 0.001), IgG (*p* = 0.005), IgA (*p* = 0.000), occult blood (*p* = 0.012), 24-h urine protein (*p* = 0.001). In addition, gender (*p* = 0.317), average Pressure (*p* = 0.198), RBC (*p* = 0.648), WBC (*p* = 0.970), HGB (*p* = 0.570), PLT (*p* = 0.409), NEUT (*p* = 0.178), ALT (*p* = 0.068), ALP (*p* = 0.145), CRE (*p* = 0.165), UA (*p* = 0.904), eGFR (*p* = 0.120), UREA (*p* = 0.265), TG (*p* = 0.128), HDL-C (*p* = 0.302), IgM (*p* = 0.340), complement C3 (*p* = 0.345)

The multivariate logistic regression model included univariate predictors associated with IgAN. Multivariate regression analysis showed that IgA (OR = 0.181, 95% CI = 0.084–0.279, *p* = 0.000) and urine occult blood (OR = 0.109, 95% CI = 0.019–0.199, *p* = 0.019) were independent predictors of IgAN in the clinical model. In the test cohort, the ROC of IgA was 0.761 (0.601–0.921), and the ROC of urine occult blood was 0.694 (0.524–0.864)

### Performance of radiomics model

**Figure 3.**
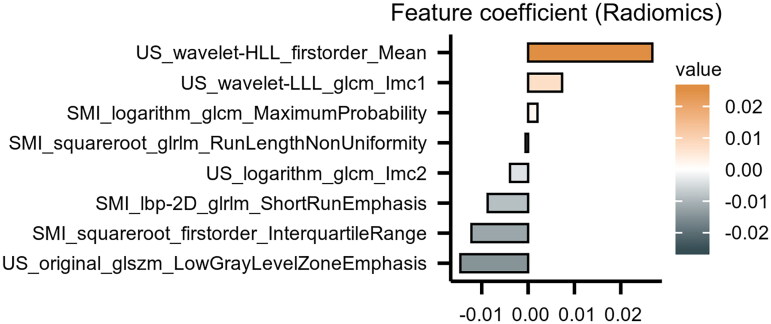
Spearman correlation coefficients were calculated for the eight selected features in Radiomics model, and their respective coefficient values. The longer x-axis variables of individual feature denoted that it played the more important role in the model.

**Figure 4.**
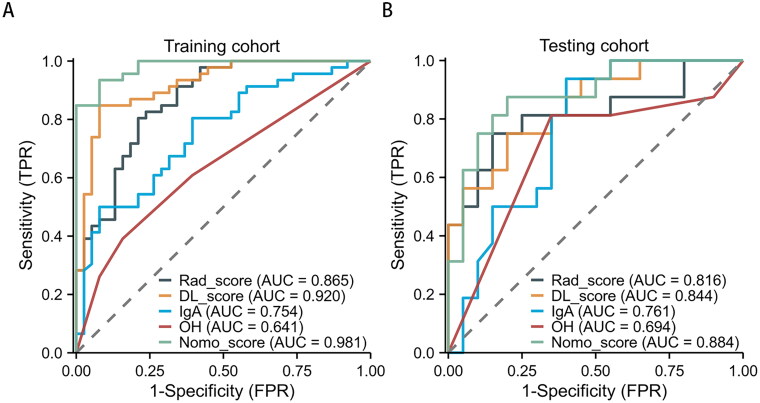
The Receiver operating characteristic (ROC) curves of the five models. (A) Five ML model ROC curves in the training cohort. (B) Three model ML ROC curves in the testing cohort.

A total of 3,384 ultrasonographic features were extracted from the ultrasound images. 1728 features were found to be significantly different between the two groups, through inter- and intra-observer analyses and the Mann-Whitney *U*-test. These significant features were further selected using LASSO, which resulted in the most optimal features set including eight radiomics features (Figure 3). The radiomics model was then established based on these features. The predictive performance of the IgAN radiomics was assessed using the ROC curve, which showed that the AUC was 0.865 for the training cohort and 0.816 for the testing cohort (Figure 4). In the training cohort, the radiomics model achieved an accuracy of 79.76%, a sensitivity of 80.44% and a specificity of 78.95% in predicting IgAN. Similarly, in the testing cohort, the accuracy, sensitivity, and specificity of the radiomics model were 77.78%, 75.00%, and 80.00%, respectively (Table 2).

**Table 2.** Performance of the three models.

Model	Group	AUC	ACC	SEN	SPE	PPV	NPV
**Radiomics**	Training cohort	0.865	79.8	80.4	79	82.2	76.9
	Testing cohort	0.816	75	80	75	80	75
**Deep Leaning**	Training cohort	0.92	88.1	84.8	92.1	92.9	83.3
	Testing cohort	0.844	69.4	75	65	63.2	76.5
**Nomogram**	Training cohort	0.981	92.9	93.5	93.5	93.5	92.1
	Testing cohort	0.884	80.6	75	85	80	81

ACC: Accuracy; SEN: sensitivity; SPE: specificity; PPV: positive predictive value;NPV: negative predictive value; AUC: area under curve.

### Performance of DL model

**Figure 5.**
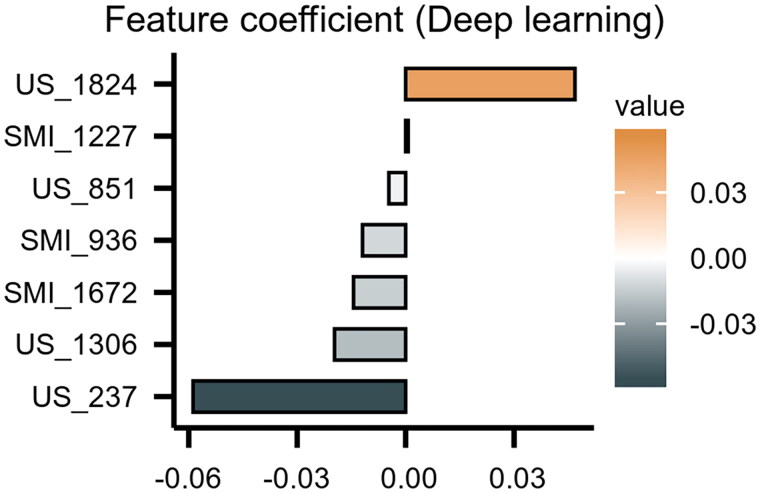
Spearman correlation coefficients were calculated for the 7 selected features in DL model, and their respective coefficient values. The longer x-axis variables of individual feature denoted that it played the more important role in the model.

Using the siamese neural network, 4096 deep learning features were extracted from each patient’s images, which included traditional US images and SMI images. Feature selection pipeline was performed using the same steps as radiomics analysis, and the most significant deep learning features were selected. Ultimately, seven deep features were selected to build the deep learning model (Figure 5). The DL_model was evaluated using the AUC, which showed that the AUCs were 0.994 and 0.844 in the training and testing cohorts, respectively (Figure 4). In the training cohort, the DL_model achieved an accuracy of 88.10%, a sensitivity of 84.78%, and a specificity of 92.11% in predicting IgAN. In the testing cohort, the accuracy, sensitivity, and specificity of the DL_model were 69.44%, 75.00%, and 65.00%, respectively (Table 2).

### Performance of clinical combinatorial nomogram model for DL radiomics

**Figure 6.**
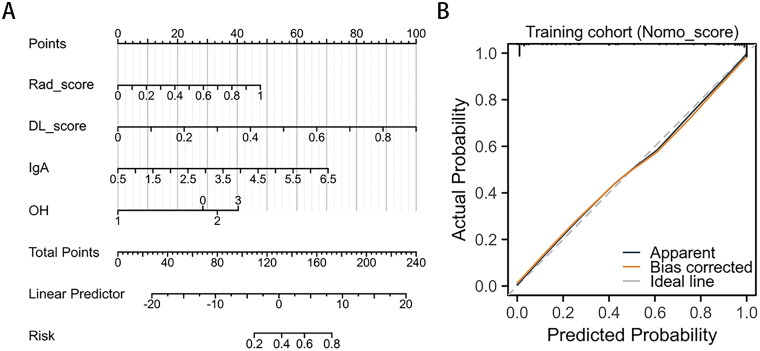
The combined nomogram model. (A) The values of DL Scorec, rad score and clinical characteristics can be converted into quantitative values according to the points axis. After summing the individual points to achieve the final sum shown on the total points axis, the evaluation of this IgA nephropathy is shown. (B) Calibration plots of nomogram for predicting IgAN.

**Figure 7.**
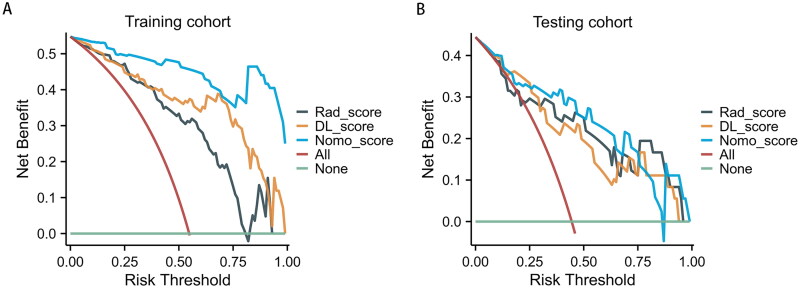
The decision curve analysis for the five model. (A) the decision curve analysis in the training cohort. (B) the decision curve analysis in the testing cohort.

We developed a combined nomogram to noninvasively differentiate IgAN and non-IgAN. The nomogram model combines five features, including DL_model score, Rad_model score, IgA, and hematuria. Each feature contributed to the nomogram output score based on a specific coefficient, and the total score of each patient was then calculated to determine the probability of IgAN. The AUC for the training and testing cohorts were 0.920 and 0.884 (Figure 4), respectively. In the training cohort, the model achieved an accuracy of 92.86%, a sensitivity of 93.48%, and a specificity of 92.11% in predicting IgAN. In the testing cohort, the accuracy, sensitivity, and specificity of the nomogram model were 80.56%, 75.00%, and 85.00%, respectively (Table 2). The calibration chart demonstrated a great consistency between the nomogram prediction probability and the actual results of IgAN (Figure 6). Additionally, the decision curve analysis revealed that the nomogram model yielded great net benefits in a risk threshold range of 0–1 in the training cohort, and a risk threshold range of 0–0.83 in testing cohort (Figure 7).

## Discussion

IgAN is the most common primary glomerular disease worldwide and the main cause of renal failure, requiring renal replacement therapy [32,33]. Early diagnosis is crucial for preventing IgAN from progressing to end-stage kidney disease. However, due to different reasons, renal biopsy is often not performed, resulting in underestimated IgAN [34]. Renal biopsy is the gold standard for diagnosing IgAN but is frequently not performed for different reasons [34]. Therefore, there is an urgent need for a simple and noninvasive IgAN diagnostic model. In this prospective study, we used DL and radiomics to extract patients’ SMI and two-dimensional ultrasound features. We combined them with clinical features to establish a nomogram model to distinguish IgAN from non-IgAN noninvasively. The model showed good discrimination in the main (AUC = 0.981) and verification (AUC = 0.884) sets. This study is the first to use a combined nomogram model based on SMI to distinguish IgAN from non-IgAN noninvasively.

Thrombotic microangiopathy is common in IgAN [33,35]. We chose SMI to reflect the kidney’s blood flow because SMI provides a lot of numerical and visual information [16,36]. Furthermore, like CEUS microflow imaging, SMI displays the vascular architecture and blood flow grade [16,37]. We extracted the image features of patients’ radiology and performed DL using these features; the results showed that the extracted image features originate from both image types, confirming that two-dimensional ultrasound and the display of renal blood flow could be potential biological markers for distinguishing between IgAN and non-IgAN. DL radiomics automatically extracts high-throughput quantitative features from medical images and then comprehensively quantifies intrarenal heterogeneity, showing better performance than single radiomics and DL methods [30]. In this study, we extracted eight radiologic features and seven DL features. These selected imaging features are not redundant but complementary.

After multivariate analysis of clinical laboratory indicators, IgA and urine occult blood were independent predictors for distinguishing IgAN from non-IgAN, consistent with previous studies [10,38]. Furthermore, the combination model we developed combines DL and radiomics predictive probability with the conventional available clinical factors. Its results are significantly better than those of the single DL and radiomics model; thus, it can be used as a translatable clinical tool that is easy to implement. For clinical use, a better result of the combination model could be obtained from decision curve analysis for most of the threshold probabilities, indicating that the combination model for treatment strategy will lead to better clinical results. Therefore, the clinical model of DL imaging based on SMI can effectively distinguish IgAN from non-IgAN, guide the treatment plan, and help implement personalized treatment.

Our research has some limitations: 1. The sample size was relatively limited, and the results may have some deviation. In future research, we will further expand the number of cases. 2. All data were from one institution, and only data from one equipment supplier were used. 3. The model has not been verified in an external independent cohort.

In conclusion, we propose a nomogram diagnosis model based on DL radiomics and clinical features displayed by SMI. This model extracts the features from SMI-based images through a convolutional neural network and fuses these features with the radiomics and clinical features to distinguish IgAN from non-IgAN. The results showed that the DL radiomics model based on SMI could effectively distinguish IgAN patients from non-IgAN patients. This model has good clinical applicability. The model provides an easy-to-use and personalized tool for noninvasive diagnosis of IgAN and can help physicians develop a more beneficial treatment plan for patients.

## Data Availability

The study data may be provided by contacting the corresponding author

## References

[1] Li LS, Liu ZH. Epidemiologic data of renal diseases from a single unit in China: analysis based on 13,519 renal biopsies. Kidney Int. 2004;66(3):1–9. doi: 10.1111/j.1523-1755.2004.00837.x. 15327382

[2] Rodrigues JC, Haas M, Reich HN. IgA nephropathy. Clin J Am Soc Nephrol. 2017;12(4):677–686. doi: 10.2215/CJN.07420716. 28159829 PMC5383386

[3] Berthoux FC, Mohey H, Afiani A. Natural history of primary IgA nephropathy. Semin Nephrol. 2008;28(1):4–9. doi: 10.1016/j.semnephrol.2007.10.001. 18222341

[4] Hricak H, Cruz C, Romanski R, et al. Renal parenchymal disease: sonographic-histologic correlation. Radiology. 1982;144(1):141–147. doi: 10.1148/radiology.144.1.7089245. 7089245

[5] Whittier WL, Korbet SM. Timing of complications in percutaneous renal biopsy. J Am Soc Nephrol. 2004;15(1):142–147. doi: 10.1097/01.asn.0000102472.37947.14. 14694166

[6] Webster AC, Nagler EV, Morton RL, et al. Chronic kidney disease. Lancet. 2017;389(10075):1238–1252. doi: 10.1016/S0140-6736(16)32064-5. 27887750

[7] Trajceska L, Severova-Andreevska G, Dzekova-Vidimliski P, et al. Complications and risks of percutaneous renal biopsy. Open Access Maced J Med Sci. 2019;7(6):992–995. doi: 10.3889/oamjms.2019.226. 30976347 PMC6454172

[8] Ren L, Zhang C, Pan Y, et al. The value of serum IgA in the diagnosis, clinical and pathological evaluation of patients with IgA nephropathy found during physical examination in China. Clin Lab. 2019;65(12):2355–2360. doi: 10.7754/Clin.Lab.2019.190444. 31850707

[9] Zhang L, Chen Z, Feng L, et al. Preliminary study on the application of renal ultrasonography radiomics in the classification of glomerulopathy. BMC Med Imaging. 2021;21(1):115. doi: 10.1186/s12880-021-00647-8. 34301205 PMC8305820

[10] Hou J, Fu S, Wang X, et al. A noninvasive artificial neural network model to predict IgA nephropathy risk in Chinese population. Sci Rep. 2022;12(1):8296. doi: 10.1038/s41598-022-11964-5. 35585099 PMC9117316

[11] Serino G, Pesce F, Sallustio F, et al. In a retrospective international study, circulating miR-148b and let-7b were found to be serum markers for detecting primary IgA nephropathy. Kidney Int. 2016;89(3):683–692. doi: 10.1038/ki.2015.333. 26581012

[12] Collaku E, Simonini R, Balbi M, et al. Superb microvascular imaging (SMI) compared with color doppler ultrasound for the assessment of hepatic artery in pediatric liver transplants: a feasibility study. Diagnostics. 2022;12(6):1476. doi: 10.3390/diagnostics12061476. PMC9221873 35741286

[13] Jeon SK, Lee JY, Kang HJ, et al. Additional value of superb microvascular imaging of ultrasound examinations to evaluate focal liver lesions. Eur J Radiol. 2022;152:110332. doi: 10.1016/j.ejrad.2022.110332. 35552066

[14] Machado P, Segal S, Lyshchik A, et al. A novel microvascular flow technique: initial results in thyroids. Ultrasound Q. 2016;32(1):67–74. doi: 10.1097/RUQ.0000000000000156. 25900162

[15] Zhu YC, Zhang Y, Shan J, et al. Added value of superb microvascular imaging and virtual touch imaging quantification in assisting thyroid cancer classification. Ultrasound Med Biol. 2021;47(12):3364–3371. doi: 10.1016/j.ultrasmedbio.2021.07.017. 34489133

[16] Mao Y, Mu J, Zhao J, et al. The comparative study of color doppler flow imaging, superb microvascular imaging, contrast-enhanced ultrasound micro flow imaging in blood flow analysis of solid renal mass. Cancer Imaging. 2022;22(1):21. doi: 10.1186/s40644-022-00458-2. 35505388 PMC9066849

[17] Meng Q, Xie X, Li L, et al. Assessment of neovascularization of carotid artery atherosclerotic plaques using superb microvascular imaging: a comparison with contrast-enhanced ultrasound imaging and histology. Quant Imaging Med Surg. 2021;11(5):1958–1969. doi: 10.21037/qims-20-933. 33936978 PMC8047364

[18] Diao XH, Shen Y, Chen L, et al. Superb microvascular imaging is as sensitive as contrast-enhanced ultrasound for detecting synovial vascularity in rheumatoid arthritis. Quant Imaging Med Surg. 2022;12(5):2866–2876. doi: 10.21037/qims-21-859. 35502398 PMC9014166

[19] Zhu Y, Meng Z, Fan X, et al. Deep learning radiomics of dual-modality ultrasound images for hierarchical diagnosis of unexplained cervical lymphadenopathy. BMC Med. 2022;20(1):269. doi: 10.1186/s12916-022-02469-z. 36008835 PMC9410737

[20] Shen YT, Chen L, Yue WW, et al. Artificial intelligence in ultrasound. Eur J Radiol. 2021;139:109717. doi: 10.1016/j.ejrad.2021.109717. 33962110

[21] Zhang N, Li XT, Ma L, et al. Application of deep learning to establish a diagnostic model of breast lesions using two-dimensional grayscale ultrasound imaging. Clin Imaging. 2021;79:56–63. doi: 10.1016/j.clinimag.2021.03.024. 33887507

[22] Lambin P, Leijenaar RTH, Deist TM, et al. Radiomics: the bridge between medical imaging and personalized medicine. Nat Rev Clin Oncol. 2017;14(12):749–762. doi: 10.1038/nrclinonc.2017.141. 28975929

[23] Bi WL, Hosny A, Schabath MB, et al. Artificial intelligence in cancer imaging: clinical challenges and applications. CA: a Cancer Journal for Clinicians. 2019;69(2):127–157. 30720861 10.3322/caac.21552 PMC6403009

[24] Qin X, Xia L, Zhu C, et al. Noninvasive evaluation of lupus nephritis activity using a radiomics machine learning model based on ultrasound. J Inflamm Res. 2023;16:433–441. doi: 10.2147/JIR.S398399. 36761904 PMC9904229

[25] Qin X, Xia L, Hu X, et al. A novel clinical-radiomic nomogram for the crescent status in IgA nephropathy. Front Endocrinol (Lausanne). 2023;14:1093452. doi: 10.3389/fendo.2023.1093452. 36742388 PMC9895811

[26] Xie X, Yang L, Zhao F, et al. A deep learning model combining multimodal radiomics, clinical and imaging features for differentiating ocular adnexal lymphoma from idiopathic orbital inflammation. Eur Radiol. 2022;32(10):6922–6932. doi: 10.1007/s00330-022-08857-6. 35674824

[27] Qin X, Zhu J, Tu Z, et al. Contrast-enhanced ultrasound with deep learning with attention mechanisms for predicting microvascular invasion in single hepatocellular carcinoma. Acad Radiol. 2023;30 (Suppl 1): S73–S80. doi: 10.1016/j.acra.2022.12.005. 36567144

[28] Ning Z, Luo J, Li Y, et al. Pattern classification for gastrointestinal stromal tumors by integration of radiomics and deep convolutional features. IEEE J Biomed Health Inform. 2019;23(3):1181–1191. doi: 10.1109/JBHI.2018.2841992. 29993591

[29] Gao W, Wang W, Song D, et al. A predictive model integrating deep and radiomics features based on gadobenate dimeglumine-enhanced MRI for postoperative early recurrence of hepatocellular carcinoma. Radiol Med. 2022;127(3):259–271. doi: 10.1007/s11547-021-01445-6. 35129757

[30] Cui Y, Zhang J, Li Z, et al. A CT-based deep learning radiomics nomogram for predicting the response to neoadjuvant chemotherapy in patients with locally advanced gastric cancer: a multicenter cohort study. EClinicalMedicine. 2022;46:101348. doi: 10.1016/j.eclinm.2022.101348. 35340629 PMC8943416

[31] He K, Zhang X, Ren S, et al. Identity mappings in deep residual networks. Paper presented at: Computer Vision–ECCV 2016: 14th European Conference, 2016 Oct 11–14; Amsterdam, The Netherlands. Proceedings, Part IV 142016.

[32] Nair R, Walker PD. Is IgA nephropathy the commonest primary glomerulopathy among young adults in the USA? Kidney Int. 2006;69(8):1455–1458. doi: 10.1038/sj.ki.5000292. 16531983

[33] Pattrapornpisut P, Avila-Casado C, Reich HN. IgA nephropathy: core curriculum 2021. Am J Kidney Dis. 2021;78(3):429–441. doi: 10.1053/j.ajkd.2021.01.024. 34247883

[34] Moresco RN, Speeckaert MM, Delanghe JR. Diagnosis and monitoring of IgA nephropathy: the role of biomarkers as an alternative to renal biopsy. Autoimmun Rev. 2015;14(10):847–853. doi: 10.1016/j.autrev.2015.05.009. 26026694

[35] El Karoui K, Hill GS, Karras A, et al. A clinicopathologic study of thrombotic microangiopathy in IgA nephropathy. J Am Soc Nephrol. 2012;23(1):137–148. doi: 10.1681/ASN.2010111130. 22052055 PMC3269921

[36] Kurt SA, Eryurekli AE, Kayadibi Y, et al. Diagnostic performance of superb microvascular imaging in differentiating benign and malignant axillary lymph nodes. Ultrasound Q. 2023;39(2):74–80. doi: 10.1097/RUQ.0000000000000617. 35943392

[37] Lan Y, Li N, Song Q, et al. Correlation and agreement between superb micro-vascular imaging and contrast-enhanced ultrasound for assessing radiofrequency ablation treatment of thyroid nodules: a preliminary study. BMC Med Imaging. 2021;21(1):175. doi: 10.1186/s12880-021-00697-y. 34809604 PMC8609811

[38] Han QX, Wang Y, Zhu HY, et al. A non-invasive diagnostic model of immunoglobulin a nephropathy and serological markers for evaluating disease severity. Chin Med J. 2019;132(6):647–652. doi: 10.1097/CM9.0000000000000121. 30855344 PMC6416104

